# Association of general psychological factors with frequent attendance in primary care: a population-based cross-sectional observational study

**DOI:** 10.1186/s12875-017-0621-5

**Published:** 2017-03-24

**Authors:** André Hajek, Jens-Oliver Bock, Hans-Helmut König

**Affiliations:** Department of Health Economics and Health Services Research, Hamburg Center for Health Economics, University Medical Center Hamburg-Eppendorf, Hamburg, Germany

**Keywords:** Primary health care, General Practitioners, Health Care Utilization, Health Services Needs and Demand

## Abstract

**Background:**

Whereas several studies have examined the association between frequent attendance in primary care and *illness-specific* psychological factors, little is known about the relation between frequent attendance and *general* psychological factors. Thus, the aim of this study was to investigate the association between being a frequent attender in primary care and general psychological factors.

**Methods:**

Data were used from a large, population-based sample of community-dwelling individuals aged 40 and above in Germany in 2014 (*n =* 7,446). Positive and negative affect, life satisfaction, optimism, self-esteem, self-efficacy, and self-regulation were included as general psychological factors. The number of self-reported GP visits in the past twelve months was used to quantify frequency of attendance; individuals with more than 9 visits (highest decile) were defined as frequent attenders.

**Results:**

Multiple logistic regressions showed that being a frequent attender was positively associated with less life satisfaction [OR: 0.79 (0.70–0.89)], higher negative affect [OR: 1.38 (1.17–1.62)], less self-efficacy [OR: 0.74 (0.63–0.86)], less self-esteem [OR: 0.65 (0.54–0.79)], less self-regulation [OR: 0.74 (0.60–0.91)], and higher perceived stress [OR: 1.46 (1.28–1.66)], after adjusting for sociodemographic factors, morbidity and lifestyle factors. However, frequent attendance was not significantly associated with positive affect and self-regulation.

**Conclusions:**

The present study highlights the association between general psychological factors and frequent attendance. As frequent GP visits produce high health care costs and are potentially associated with increased referrals and use of secondary health care services, this knowledge might help to address these individuals with high needs.

**Electronic supplementary material:**

The online version of this article (doi:10.1186/s12875-017-0621-5) contains supplementary material, which is available to authorized users.

## Background

Only a small proportion of patients account for large number of encounters to general practices [1]. Since these frequent attenders consume a large share of health care resources [2], many studies have tried to characterize them, particularly focusing on certain physical, psychological and social factors [3]. This cited literature review found, for example, a high illness level, low social support, unemployment or divorce to be associated with frequent attendance to general practices. Thereby, analyses of psychological factors were restricted to mental health disorders or, more generally, mental problems, which were an equally important factor for frequent attendance [3].

In general, studies analyzing health care use or, more specifically, frequent attendance often base their analysis on Andersen’s Behavioral model. This model distinguishes predisposing factors, like general demographics, enabling resources like income or health insurance, and need factors such as perceived and evaluated illness level as determinants of health care use [4].

This model has been modified several times including a version for health care use of vulnerable people [5]. The modified version of the model also refers to psychological factors as predisposing factors and include constructs, such as coping or self-esteem. Yet, despite that, little is known about the association of general psychological factors and frequent attendance in primary care. This is despite the fact that some previous studies indicated that general psychological factors, like satisfaction with life, self-esteem, self-efficacy, etc., might play an important role for a better understanding of frequent attendance in primary care. Thus, for example, a cross-sectional study showed that frequent attenders (109 frequent attenders at a general practice surgery) differed substantially compared to a control group (86 individuals) with regard to the personality trait of neuroticism [6]. Furthermore, another cross-sectional observational study (suburban teaching practice with 12,400 patients, UK) showed that frequent attenders (*n =* 132 patients) and controls (*n =* 102 age and sex-matched control patients) differed significantly in all considered dimensions of well-being, such as energy, emotional reactions or social isolation [7]. Another psychological construct associated with frequent attendance is perceived susceptibility to and severity of illnesses. These findings were made by a cross-sectional observational study conducted in Spain (public Health Centre in Granada, 236 frequent attenders and 420 controls, matched by sex and age) [8].

In addition, in a case control study (100 regular and 100 normal attenders; England) it has been shown that one reason of frequent attendance in primary care could be somatization [9], which is the presence of somatic complaints that cannot be explained by organic findings [10]. This stresses the importance of psychological dimensions of frequent attendance, as somatization is highly associated with psychological factors, such as negative affect or illness behavior [11]. More generally, as stated in a literature review, because different persons perceive identical organic symptoms completely differently, consultation rates do not only rely on need factors, but moreover on the way a person perceives his body and arising symptoms [12]. This means that frequent attenders might be characterized by certain character traits or general psychological constructs.

However, *comprehensive* investigations of well-established psychological concepts with regard to frequent attendance are missing. Therefore, the aim of this study was to determine associations of the well-established psychological factors of life satisfaction, positive and negative affect, optimism, self-efficacy, self-esteem, self-regulation, as well as perceived stress with frequent attendance in a population-based sample of Germans aged 40 and older. Consequently, the present study adds new insights into the relation between general psychological factors and frequent attendance. Unlike other studies, general psychological factors were used and data were gathered from a large representative sample of individuals in the second half of life. Knowing psychological factors associated with frequent attendance might help to address these individuals with high needs.

Subjective well-being (positive and negative affect, and life satisfaction) were included in our study because it has been demonstrated that it is associated with health-related behavior [13], which in turn is related to frequent attendance. Furthermore, it has been shown that stress [14] as well as optimism [15] are positively associated with neuroticism, which is known to be associated with an increase in health care use [16]. Therefore, these factors were included in our study. In addition, it has been found that optimism is related to physical and mental health [17], which is associated with frequent attendance [18]. Furthermore, it appears plausible that self-regulation is related to frequent attendance because individuals scoring high in self-regulation might be more willing to visit GP more often in order to achieve the goal of good health. Besides, individuals scoring high in self-efficacy believe in their skills to achieve goals. Consequently, they might believe that frequent physician visits are not required to reach their goals. Self-esteem was included in our study because individuals scoring low in self-esteem tend to be more anxious and had poor physical functioning [19] – factors that are associated with an increased likelihood of frequent attendance [20, 21].

## Methods

### Sample

Cross-sectional data were derived from the fifth wave (2014) of the German Ageing Survey (DEAS), funded by the Federal Ministry for Family Affairs, Senior Citizens, Women and Youth (BMFSFJ). The DEAS study is a representative survey among community-dwelling older adults (aged 40 and above). Since 1996, data were collected about social exclusion, income, occupation, health and various other issues. While more than 4,000 individuals have already been interviewed in former waves (response rate: 61%), about 6,000 participants from the birth cohorts 1929 to 1974 were interviewed for the first time in the fifth wave (response rate: 25%). In wave 5, 7,446 individuals filled out the drop-off questionnaire and delivered data on GP visits. Klaus and Engstler [22] provided more details with regard to the fifth wave as well as the sampling frame and sample composition. We focused on the fifth wave of the DEAS study as our main interest lay in investigating the association between frequent attendance and psychological factors such as self-regulation and perceived stress, which were only collected in this wave. Moreover, the exact number for all GP visits were solely reported in the fifth wave.

### Dependent variable

To measure the number of GP visits in the drop-off questionnaire of the German Ageing Survey, individuals were asked whether (no; yes) and, if so, how often they had visited GPs in the past twelve months. In this questionnaire, the individuals should include house calls. Solely collecting a prescription was not considered as a visit in the drop-off questionnaire.

In main analysis, frequent attendance (=1) was defined as the 10% of patients with most frequent contacts (i.e. the number of GP visits was ≥ 10 in our study). This definition is in accordance with many previous studies [3].

To test the robustness of our results, highest quartile (GP visits ≥ 5) and the highest 5% (GP visits ≥ 12) were also used to quantify frequent attendance in additional analysis.

### Independent variables: psychological variables

The widely used and well-validated [23] positive and negative affect schedule (PANAS) [24] was used to measure negative affect (emotions such as anger or fear) and positive affect (emotions such as joy or excitement), consisting of ten items (ranging from 1 = “very slightly or not at all” to 5 =”extremely”). The index score (1–5) for positive affect as well as negative affect was computed by averaging the score of the corresponding items (higher values reflect higher positive affect/negative affect; Cronbach’s Alpha for the PANAS was .87).

The Satisfaction with Life Scale (SWLS) [25] which has very good psychometric properties [26] was used to assess life satisfaction (reflecting the cognitive evaluation of one’s life as a whole). The SWLS consists of five items (ranging from 1 = “strongly agree” to 5 = “strongly disagree”). The higher the index score (1-5) is, the higher is the life satisfaction (Cronbach’s Alpha was .86).

Brandstädter and Wentura [27] created the validated optimism scale (reflecting the perspectives about the future). The scale comprises five items, each ranging from 1 = “strongly agree” to 4 = “strongly disagree”. An index score (1–4) was created by averaging the items (higher values representing higher optimism; Cronbach’s Alpha was .84).

Perceived stress (reflecting the degree to which situations in one’s life are considered as stressful) was quantified by using a scale which was developed by Cohen et al. [28]. The scale covers four items and the index score ranges from 1 to 5 (higher values represent higher perceived stress; Cronbach’s Alpha was .70).

Schwarzer and Jerusalem [29] developed a scale to quantify self-efficacy (reflecting the ability that an individual has to do a certain action), with five items ranging from 1 = “strongly agree” to 4 = “strongly disagree”. The higher the index score (1-4) is, the higher is the self-efficacy (Cronbach’s Alpha was .75).

Based on the theory of selection, optimization, and compensation (SOC), self-regulation (reflecting the ability to withstand competing attractions) was assessed. The validated scale [30] has four items (1-5). The index value (1-5) was generated by averaging the items. Higher values correspond to higher self-regulation (Cronbach’s Alpha was .78).

Self-esteem (reflecting the conscious feeling of self-worth and acceptance) was measured by the widely used and validated Rosenberg scale [31] which consists of ten items (from 1 = “strongly agree” to 4 = “strongly disagree”). An index score (1-4) was computed by averaging the items, with higher values reflecting higher self-esteem (Cronbach’s Alpha was .84).

### Independent variables: control variables

In addition to psychological variables, control variables were included, namely age, family status (married, living together with spouse, and others (married, living separated from spouse; divorced; widowed; never married), as well as employment status (working; retired; not employed). Furthermore, the region (West and East Germany), and individual log monthly net equivalence income in Euro (new OECD scale) was included. In addition, the number of physical illnesses (yes; no) such as cancer, diabetes, bladder problems, bad circulation or insomnia was included to measure morbidity (ranging from 0 to 11). Four lifestyle factors were also included. The days with alcohol consumption (e.g., spirits, long drinks, wine or beer) was used (daily alcohol consumption, and others (several times a week, once a week, 1 to 3 times a month, less often and never)). In addition, the smoking status (no; yes) and self-reported Body Mass Index (BMI) was included. Furthermore, physical activities such as hiking, soccer, swimming or gymnastics were assessed (at least once a week (daily; several times a week; once a week) vs. less than once a week (1-3 times a month; less often)).

There is evidence that frequent attendance in primary care is associated with depression.[7, 20, 21] Consequently, in additional analysis, the main model was extended by adding depression (1 if Center for Epidemiological Studies Depression Scale (15 items, 0-45) ≥ 18 [32]) to test the robustness of our results. Depression was omitted in the main models since it is correlated with the psychological factors.

### Statistical analysis

First, bivariate comparisons between non-frequent attenders and frequent attenders were conducted using Chi^2^-tests and independent t-tests for categorical and continuous data, respectively. Second, multiple logistic regressions were performed. The criterion for statistical significance was set at *p <* .05. All analyses were conducted using Stata 14.0 (StataCorp, College Station, Texas, USA). Since the general psychological factors are strongly associated with each other, they were included separately in the regression models. More importantly, the main motivation why the general psychological factors were included separately in the regression models was to show that whenever one of these general psychological variables is available, future studies of frequent attendance should include this factor as explanatory variable. This is because we hypothesized that these factors are significantly associated with frequent attendance, after adjustment for a full set of potential confounders (e.g., sociodemographic variables, lifestyle factors and morbidity).

## Results

### Bivariate associations

Mean GP visits were 3.9 (±5.2), ranging from 0 to 120 visits. Median GP visits were 3. The highest quartile had at least 5 GP visits, whereas the highest decile had 10 or more GP visits. In addition, the top 5% had 12 or more GP visits (these results are not shown in Table 1).

**Table 1:** Sample characteristics, by status (non-frequent attenders vs. frequent attenders) (*n =* 7,446)

Variables	Non-frequent attenders (*n =* 6,700)	Frequent attenders (*n =* 746)	*p*-value	Missings (%)
Female (Ref.: Male): N (%)	3,385 (50.5)	399 (53.5)	ns	0.0
Age in years: Mean (SD); Range	64.1 (11.2); 40–95	67.8 (11.3); 40–93	<.001	0.0
Employment status: N (%)			<.001	0.0
Working	2,576 (38.5)	150 (20.1)		
Retired	3,549 (53.0)	505 (67.7)		
Not employed	572 (8.5)	91 (12.2)		
Married, living together with spouse (Ref.: Others): N (%)	4,745 (70.9)	460 (62.0)	<.001	0.2
Monthly net equivalence income in Euro: Mean (SD); Range	1,976.2 (1,415.1); 80–33,333.3	1,579.9 (828.3); 122.0–7,018.0	<.001	5.3
East Germany (Ref.: West Germany): N (%)	2,238 (33.4)	228 (30.6)	ns	0.0
Sports at least once a week (Ref.: Sports less than once a week): N (%)	3,693 (55.1)	331 (44.4)	<.001	0.0
Body-Mass-Index (BMI): Mean (SD); Range	26.7 (4.4); 13.2–55.8	28.5 (5.5); 16.4–60.9	<.001	1.7
Current smoker (Ref.: No): N (%)	1,178 (17.7)	122 (16.5)	ns	0.8
Daily alcohol consumption (Ref.: Less than daily alcohol consumption): N (%)	818 (12.2)	70 (9.4)	<.05	0.2
Number of physical illnesses: Mean (SD); Range	2.5 (1.8); 0–11	4.0 (2.0); 0–11	<.001	1.5
Life satisfaction (Pavot/Diener 1993): Mean (SD); Range	3.8 (0.7); 1–5	3.5 (0.9); 1–5	<.001	0.9
Positive affect (Watson, Clark & Tellegen, 1988): Mean (SD); Range	3.6 (0.5); 1–5	3.4 (0.5); 1.4–5	<.001	0.9
Negative affect (Watson, Clark & Tellegen, 1988): Mean (SD); Range	2.1 (0.5); 1–5	2.2 (0.6); 1–4.6	<.001	0.9
Optimism (Brandstädter & Wentura, 1994): Mean (SD); Range	3.0 (0.5); 1–4	2.8 (0.6); 1–4	<.001	0.3
Self-efficacy (Schwarzer & Jerusalem, 1999): Mean (SD); Range	3.1 (0.4); 1–4	2.9 (0.5); 1-4	<.001	0.4
Self-esteem (Rosenberg 1965): Mean (SD); Range	3.4 (0.4); 1.4–4	3.3 (0.5); 1.2–4	<.001	0.1
Self-regulation (Freund & Baltes, 2002): Mean (SD); Range	4.0 (0.5); 2-5	3.9 (0.5); 2–5	<.01	2.0
Perceived stress (Cohen et al., 1983): Mean (SD); Range	2.3 (0.6); 1–5	2.6 (0.7); 1–4.8	<.001	1.5

*ns* not significant, *N* number, *SD* standard deviation; Comparisons between the two groups were done using *t*-test and chi-square procedures

Sample characteristics are displayed in Table 1, separated by status (non-frequent attenders vs. frequent attenders). Compared to non-frequent attenders, frequent attenders (= highest decile in GP visits) had a significant higher age, a lower income, a higher BMI and a higher number of physical illnesses. Moreover, frequent attendance was significantly associated with employment status, marital status, sports, and alcohol consumption, whereas it was not significantly associated with sex, region, and smoking status. Furthermore, frequent attenders had a lower satisfaction with life, less positive affect, higher negative affect, less optimism, less self-efficacy, less self-esteem, less self-regulation and higher perceived stress.

Furthermore, it is worth noting that psychological variables were associated from r = -.22 (self-regulation and negative affect as well as self-regulation and social exclusion) to .61 (life satisfaction and optimism as well as self-esteem and optimism).

### Regression analysis: main analysis

The results of multiple logistic regressions (determinants of frequent attendance) are displayed in Table 2 (highest decile; main model), Table 3 (highest quartile; additional analysis) and Table 4 (highest 5%; additional analysis). In all multiple logistic regressions, we adjusted for age, (log) equivalence income, number of chronic diseases, BMI, as well as dummy-variables for sex, marital status, employment status, region, sports, alcohol consumption and smoking status.

**Table 2:** Predictors of frequent attenders (0 = Non-frequent attenders; 1 = Frequent attenders; cut-off at the highest decile). Results of multiple logistic regressions^a^

	(1)	(2)	(3)	(4)	(5)	(6)	(7)	(8)
Independent variables	Frequent attenders	Frequent attenders	Frequent attenders	Frequent attenders	Frequent attenders	Frequent attenders	Frequent attenders	Frequent attenders
Potential confounders	✓	✓	✓	✓	✓	✓	✓	✓
Life satisfaction	0.79***							
	(0.70 – 0.89)							
Positive affect		0.87+						
		(0.73 – 1.02)						
Negative affect			1.38***					
			(1.17 – 1.62)					
Optimism				0.74***				
				(0.63 – 0.86)				
Self-efficacy					0.65***			
					(0.54 – 0.79)			
Self-esteem						0.74**		
						(0.60 – 0.91)		
Self-regulation							0.93	
							(0.79 – 1.10)	
Perceived stress								1.46***
								(1.28 – 1.66)
Constant	0.24+	0.34	0.07**	0.45	0.56	0.46	0.30	0.05***
	(0.05 – 1.19)	(0.06 – 1.75)	(0.01 – 0.41)	(0.09 – 2.24)	(0.11 – 2.92)	(0.09 – 2.42)	(0.06 – 1.59)	(0.01 – 0.25)
Observations	6,730	6,725	6,724	6,764	6,760	6,778	6,662	6,692
Pseudo R^2^	0.110	0.107	0.110	0.110	0.111	0.107	0.104	0.113

^a^All estimations include age, (log) equivalence income, number of chronic diseases, Body-Mass-Index, as well as dummy-variables for sex, marital status, employment status, region, sports, alcohol consumption and smoking status as potential confounders. Odds ratios were reported; 95% confidence intervals in parentheses; ***p < 0.001, **p < 0.01, *p < 0.05, + *p <* 0.10. Life satisfaction (SWLS, Pavot & Diener, 1993); Positive and negative affect (PANAS, Watson et al., 1988); Optimism (Brandtstädter & Wentura, 1994); Self-efficacy (Schwarzer & Jerusalem, 1999); Self-esteem (Rosenberg, 1965); Self-regulation (Freund & Baltes, 2002); Perceived stress (Cohen et al., 1983). The Wald test was used to test the significance of each parameter

**Table 3:** Predictors of frequent attenders (0 = Non-frequent attenders; 1 = Frequent attenders; cut-off at the highest quartile). Results of multiple logistic regressions^a^

	(1)	(2)	(3)	(4)	(5)	(6)	(7)	(8)
Independent variables	Frequent attenders	Frequent attenders	Frequent attenders	Frequent attenders	Frequent attenders	Frequent attenders	Frequent attenders	Frequent attenders
Potential confounders	✓	✓	✓	✓	✓	✓	✓	✓
Life satisfaction	0.77***							
	(0.71 – 0.84)							
Positive affect		0.74***						
		(0.66 – 0.84)						
Negative affect			1.40***					
			(1.24 – 1.58)					
Optimism				0.70***				
				(0.63 – 0.78)				
Self-efficacy					0.64***			
					(0.55 – 0.73)			
Self-esteem						0.74***		
						(0.64 – 0.86)		
Self-regulation							0.82***	
							(0.73 – 0.92)	
Perceived stress								1.48***
								(1.35 – 1.63)
Constant	0.46	0.87	0.13***	0.86	1.16	0.85	0.73	0.09***
	(0.15 – 1.47)	(0.26 – 2.85)	(0.04 – 0.43)	(0.27 – 2.76)	(0.35 – 3.81)	(0.26 – 2.79)	(0.22 – 2.45)	(0.03 – 0.30)
Observations	6,730	6,725	6,724	6,764	6,760	6,778	6,662	6,692
Pseudo R^2^	0.109	0.106	0.107	0.108	0.108	0.104	0.104	0.112

^a^All estimations include age, (log) equivalence income, number of chronic diseases, Body-Mass-Index, as well as dummy-variables for sex, marital status, employment status, region, sports, alcohol consumption and smoking status as potential confounders. Odds ratios were reported; 95% confidence intervals in parentheses; ***p < 0.001, **p < 0.01, *p < 0.05, + *p <* 0.10. Life satisfaction (SWLS, Pavot & Diener, 1993); Positive and negative affect (PANAS, Watson et al., 1988); Optimism (Brandtstädter & Wentura, 1994); Self-efficacy (Schwarzer & Jerusalem, 1999); Self-esteem (Rosenberg, 1965); Self-regulation (Freund & Baltes, 2002); Perceived stress (Cohen et al., 1983). The Wald test was used to test the significance of each parameter

**Table 4:** Predictors of frequent attenders (0 = Non-frequent attenders; 1 = Frequent attenders; cut-off at the highest 5%). Results of multiple logistic regressions^a^

	(1)	(2)	(3)	(4)	(5)	(6)	(7)	(8)
Independent variables	Frequent attenders	Frequent attenders	Frequent attenders	Frequent attenders	Frequent attenders	Frequent attenders	Frequent attenders	Frequent attenders
Potential confounders	✓	✓	✓	✓	✓	✓	✓	✓
Life satisfaction	0.79***							
	(0.68 – 0.91)							
Positive affect		0.83+						
		(0.68 – 1.02)						
Negative affect			1.34**					
			(1.10 – 1.63)					
Optimism				0.74**				
				(0.62 – 0.89)				
Self-efficacy					0.71**			
					(0.56 – 0.89)			
Self-esteem						0.81+		
						(0.63 – 1.03)		
Self-regulation							1.05	
							(0.86 – 1.28)	
Perceived stress								1.37***
								(1.18 – 1.61)
Constant	0.14*	0.21	0.05**	0.26	0.33	0.23	0.13*	0.04**
	(0.02 – 0.94)	(0.03 – 1.58)	(0.01 – 0.37)	(0.04 – 1.79)	(0.05 – 2.37)	(0.03 – 1.72)	(0.02 – 1.00)	(0.00 – 0.29)
Observations	6,730	6,725	6,724	6,764	6,760	6,778	6,662	6,692
Pseudo R^2^	0.124	0.121	0.123	0.123	0.123	0.120	0.118	0.125

^a^All estimations include age, (log) equivalence income, number of chronic diseases, Body-Mass-Index, as well as dummy-variables for sex, marital status, employment status, region, sports, alcohol consumption and smoking status as potential confounders. Odds ratios were reported; 95% confidence intervals in parentheses; ***p < 0.001, **p < 0.01, *p < 0.05, + *p <* 0.10. Life satisfaction (SWLS, Pavot & Diener, 1993); Positive and negative affect (PANAS, Watson et al., 1988); Optimism (Brandtstädter & Wentura, 1994); Self-efficacy (Schwarzer & Jerusalem, 1999); Self-esteem (Rosenberg, 1965); Self-regulation (Freund & Baltes, 2002); Perceived stress (Cohen et al., 1983). The Wald test was used to test the significance of each parameter

Adjusting for several potential confounders, logistic regressions revealed that frequent attendance was associated with less life satisfaction [OR: 0.79 (0.70–0.89)], higher negative affect [OR: 1.38 (1.17–1.62)], less self-efficacy [OR: 0.74 (0.63–0.86)], less self-esteem [OR: 0.65 (0.54–0.79)], less self-regulation [OR: 0.74 (0.60–0.91)], and higher perceived stress [OR: 1.46 (1.28–1.66)], whereas the outcome measure was not significantly associated with positive affect and self-regulation.

When general psychological factors were *not* included in the regression model (i.e. only controlling for potential confounders noted above), it is worth noting that Pseudo R^2^ was .106 (main model: highest decile), .102 (first additional model: highest quartile), and .119 (second additional model: highest 5%), respectively. When general psychological factors were included in the respective regression model, Pseudo R^2^ values can be found in the Tables 2 to 4.

### Regression analysis: additional analysis

Compared with our main model (Table 2), our additional models (Table 3; highest quartile) showed similar results in terms of significance and effect sizes. Contrarily, the association between being a frequent attender and positive affect as well as self-regulation became significant in this model specification. Furthermore, our additional models with a cut-off at the highest 5% (Table 4) showed similar results when compared with our main model. However, the association between self-esteem and the outcome measure became insignificant.

Moreover, our main model was extended by additionally controlling for depression. In terms of effect sizes and significance, findings were similar (please see Additional file 1, Additional file 2 and Additional file 3). However, the association between self-esteem and the outcome measure vanished.

## Discussion

The present study aimed at investigating the association between frequent attendance in primary care and general psychological factors in a large, population-based sample of community-dwelling individuals aged 40 and over in Germany. In the main model (10%), negative affect is positively associated with frequent attendance, while positive affect is only close to reach the level of statistical significance. Negative affect is more closely related to, for example, physical complaints and perceived stress [33]. In addition, negative affect is associated with anxiety and depression, whereas positive affect with anxiety only [34]. Furthermore, it has been shown that satisfaction with life is associated with health-related behavior [13], which is linked to frequent attendance. Hence, our results appear plausible.

In our study, frequent attendance in primary care was positively associated with increased perceived stress and a decreased optimism. Both results might be explained by the fact that stress [14] as well as optimism [15] is positively associated with neuroticism which is related to an increased health care use [16]. Moreover, optimism generally affects physical and mental health [17], with an inferior mental health status being known to be a key characteristic of frequent attenders [18]. Yet as associations of optimism and frequent attendance persisted after controlling for depression status, considering optimism adds insights into the psychological characteristics of frequent attenders as an independent factor.

In our main model, self-regulation was not associated with frequent attendance. A possible explanation might be that individuals scoring high in self-regulation do *not* believe in the fact that *frequent* doctor visits are important for their functional status. Nevertheless, doctor visits and self-regulation might be correlated to some extent because patients with higher self-regulation might be more willing to visit the GP more often to satisfy the long-term goal of staying healthy. Yet, at high levels of physician visits, this association apparently disappears.

Frequent attendance in primary care was negatively associated with self-efficacy in our study. This appears plausible because individuals with high self-efficacy believe in their *own* capabilities to achieve aims. Thus, these individuals might think that frequent doctor visits are not necessary to stay healthy. In addition, frequent attendance in primary care was negatively associated with self-esteem in our study. This might be explained by the fact that individuals with a low self-esteem tend to be, e.g., more anxious, more maladjusted or had poor functioning [19]. These factors are in turn associated with frequent GP visits [20, 21]. In sum, while previous studies focused on (illness-) specific psychological factors, the current study extends this literature by a broader perspective on general psychological factors characterizing frequent attendance in primary care.

A major strength of our study was that data were gathered from a large, population-based study of community-dwelling individuals aged 40 and above in Germany. Furthermore, the general psychological factors were quantified using well-validated scales. In addition, we used different cut-offs to quantify frequent attendance, revealing similar results. Consequently, our results were robust against the definition of frequent attenders. However, this is a cross-sectional study and therefore the temporal relationship between psychological factors and the outcome measure cannot be assessed. In our study, self-reported GP visits in the past 12 months were used. Thus, a recall bias cannot be ruled out. Given the long recall period used in the current study, under-reporting is likely to occur. If this inaccuracy is associated with variables of interest (e.g., life satisfaction or negative affect), this bias might be somewhat stronger. However, it remains an open question whether variables of interest are associated with recall of GP visits in our study. In addition, generally speaking, this recall bias might be rather small [35]. However, future studies should use, as far as data are available, objective measures of health care attendance. Moreover, sample selection bias is a limiting factor of this study. Thus, it might be difficult to generalize our findings to individuals with subjective poor skills in the German language and individuals with low education. However, in total, the sample selection bias is supposed to be rather small [36]. In addition, our findings might not be generalizable to individuals living in institutional settings.

## Conclusions

Beyond various characteristics related to the domain of in particular mental and physical health, frequent attenders of primary care setting can be characterized by various well-established psychological constructs. As frequent GP visits produce high health care costs and are potentially associated with increased referrals and the use of secondary health care services [37], this knowledge might help to address these individuals with high needs. Specific interventions are available to improve the provision of care for or to decrease the service use of these high users [38, 39]. Future interventions might additionally benefit from the insights into their psychological constitution and can identify these specific target groups better.

## Additional files

Additional file 1: Table S1. Predictors of frequent attenders (0 = Non-frequent attenders; 1 = Frequent attenders; cut-off at the highest decile). Results of multiple logistic regressions. (DOCX 14 kb)

Additional file 2: Table S2. Predictors of frequent attenders (0 = Non-frequent attenders; 1 = Frequent attenders; cut-off at the highest quartile). Results of multiple logistic regressions. (DOCX 14 kb)

Additional file 3: Table S3. Predictors of frequent attenders (0 = Non-frequent attenders; 1 = Frequent attenders; cut-off at the highest 5%). Results of multiple logistic regressions. (DOCX 14 kb)

## Abbreviations

BMFSFJ: Federal ministry for family affairs senior citizens women and youth
BMI: Body mass index
DEAS: German ageing survey
GP: General practitioner
OECD: Organization for economic Co-operation and development
PANAS: positive and negative affect schedule
SOC: selection, optimization, and compensation
SWLS: Satisfaction with life scale
