# Supplementary material for: Association of general psychological factors with frequent attendance in primary care: a population-based cross-sectional observational study
Source: BMC Fam Pract. 2017 Mar 24;18:48. doi: 10.1186/s12875-017-0621-5 (PMC5366110; doi:10.1186/s12875-017-0621-5)
Supplement: Supplementary file 1 — Predictors of frequent attenders (0 = Non-frequent attenders; 1 = Frequent attenders; cut-off at the highest decile). Results of multiple logistic regressions. (DOCX 14 kb) [file 12875_2017_621_MOESM1_ESM.docx]

Additional Table 1. Predictors of frequent attenders (0 = Non-frequent attenders; 1 = Frequent attenders; cut-off at the highest decile). Results of multiple logistic regressions.^1^

|  | (1) | (2) | (3) | (4) | (5) | (6) | (7) | (8) |
| --- | --- | --- | --- | --- | --- | --- | --- | --- |
| Independent variables | Frequent attenders | Frequent attenders | Frequent attenders | Frequent attenders | Frequent attenders | Frequent attenders | Frequent attenders | Frequent attenders |
|  |  |  |  |  |  |  |  |  |
| Potential confounders | 🗸 | 🗸 | 🗸 | 🗸 | 🗸 | 🗸 | 🗸 | 🗸 |
|  |  |  |  |  |  |  |  |  |
| Life satisfaction | 0.84** |  |  |  |  |  |  |  |
|  | (0.75 - 0.95) |  |  |  |  |  |  |  |
| Positive affect |  | 0.94 |  |  |  |  |  |  |
|  |  | (0.80 - 1.12) |  |  |  |  |  |  |
| Negative affect |  |  | 1.22* |  |  |  |  |  |
|  |  |  | (1.02 - 1.45) |  |  |  |  |  |
| Optimism |  |  |  | 0.81** |  |  |  |  |
|  |  |  |  | (0.69 - 0.95) |  |  |  |  |
| Self-efficacy |  |  |  |  | 0.71*** |  |  |  |
|  |  |  |  |  | (0.58 - 0.86) |  |  |  |
| Self-esteem |  |  |  |  |  | 0.83+ |  |  |
|  |  |  |  |  |  | (0.67 - 1.03) |  |  |
| Self-regulation |  |  |  |  |  |  | 0.97 |  |
|  |  |  |  |  |  |  | (0.82 - 1.14) |  |
| Perceived stress |  |  |  |  |  |  |  | 1.34*** |
|  |  |  |  |  |  |  |  | (1.17 - 1.54) |
| Constant | 0.16* | 0.18* | 0.08** | 0.26 | 0.32 | 0.23+ | 0.18* | 0.05*** |
|  | (0.03 - 0.83) | (0.03 - 0.97) | (0.01 - 0.45) | (0.05 - 1.37) | (0.06 - 1.70) | (0.04 - 1.28) | (0.03 - 0.97) | (0.01 - 0.27) |
|  |  |  |  |  |  |  |  |  |
| Observations | 6,630 | 6,626 | 6,625 | 6,663 | 6,659 | 6,677 | 6,565 | 6,593 |
| Pseudo R² | 0.115 | 0.113 | 0.114 | 0.115 | 0.116 | 0.113 | 0.111 | 0.116 |

^1^ All estimations include age, (log) equivalence income, number of chronic diseases, Body-Mass-Index, as well as dummy-variables for sex, depression, marital status, employment status, region, sports, alcohol consumption and smoking status as potential confounders. Odds ratios were reported; 95% confidence intervals in parentheses; *** p<0.001, ** p<0.01, * p<0.05, + p<0.10. Life satisfaction (SWLS, Pavot & Diener, 1993); Positive and negative affect (PANAS, Watson et al., 1988); Optimism (Brandtstädter & Wentura, 1994); Self-efficacy (Schwarzer & Jerusalem, 1999); Self-esteem (Rosenberg, 1965); Self-regulation (Freund & Baltes, 2002); Perceived stress (Cohen et al., 1983). The Wald test was used to test the significance of each parameter.
